# Arterial Blood-Flow Acceleration Time on Doppler Ultrasound Waveforms: What Are We Talking About?

**DOI:** 10.3390/jcm12031097

**Published:** 2023-01-31

**Authors:** Jean-Eudes Trihan, Guillaume Mahé, Jean-Pierre Laroche, Michel Dauzat, Antonia Perez-Martin, Magali Croquette, Damien Lanéelle

**Affiliations:** 1 Vascular Medicine, Cholet Hospital, 49300 Cholet, France; 2 Vascular Medicine, Angers University Hospital, 49000 Angers, France; 3 M2S—EA 7470, Rennes University, 35000 Rennes, France; 4 Vascular Medicine, Rennes University Hospital, 35033 Rennes, France; 5 Clinical Investigation Center, INSERM CIC 1414, 35033 Rennes, France; 6 Vascular Medicine, Montpellier University Hospital, 34000 Montpellier, France; 7 Medipole, 84000 Avignon, France; 8 Vascular Medicine, Nîmes University Hospital, 30900 Nîmes, France; 9 IDESP, Montpellier University, INSERM, 34000 Montpellier, France; 10 Vascular Medicine, Poitiers University Hospital, 86000 Poitiers, France; 11 Vascular Medicine, Côte de Nacre University Hospital, 14033 Caen, France; 12 COMETE Laboratory, INSERM 1075, Université de Caen, 14000 Caen, France

**Keywords:** acceleration time, maximal acceleration, systolic rise time, peripheral artery disease, lower-extremity artery disease, doppler ultrasound

## Abstract

In recent years, the assessment of systolic acceleration in lower-extremity peripheral artery disease (PAD) has been brought back into the spotlight, whatever measure is used: time (in s) or acceleration (in cm.s^−2^). Acceleration time (also called systolic rise time) and maximal acceleration are two different but very useful measurements of growing interest in PAD. A background of the historical development, physics rationale, semantics, and methods of measurement, as well as their strengths and weaknesses, are discussed herein. Acceleration time is a powerful tool for predicting significant arterial stenosis or for estimating the overall impact of PAD as it is highly correlated to the ankle or toe pressure indexes. It could even become a new diagnostic criterion for critical limb ischemia. Similarly, maximal systolic acceleration ratios are highly predictive of carotid or renal stenosis. However, the literature lacks reference standards or guidelines for the assessment of such variables, and their measurement techniques seem to differ between authors. We propose herein a semantic and measurement statement order to clarify and help standardize future research.

## 1. Rationale and Semantics

In recent years, a growing interest has emerged, or re-emerged, in assessing the severity of lower-extremity peripheral artery disease (PAD) using a Doppler ultrasound, particularly by assessing the systolic blood-flow acceleration on Doppler time–velocity waveforms (DW) from distal arteries of the foot.

Arterial stenosis and obstruction result in the downstream alteration of blood-flow velocity waveforms with a decreased mean blood-flow velocity, reduced flow pulsatility, and dampened systolic peak resulting from slowed-down systolic flow acceleration [1]. These changes can be evidenced and quantified by measuring either the duration of the systolic upslope or the systolic acceleration itself. However, the terms used to name the corresponding variables are sometimes confusing. For instance, the systolic rise time [2], (also called the acceleration time [3] or systolic upslope time [4]) does not measure acceleration itself but the time duration of the rising part of the systolic peak. This duration, reported in milliseconds (ms), does depend on early systolic acceleration, which is related to left ventricle contractility and aortic wall distensibility as well as many other factors, including heart rate, peripheral arterial wall stiffness and viscosity, and the amplitude and timing of reflected pulse waves [1,5,6]. Peripheral pulse wave reflection and aortic wall distensibility exert a combined influence on the flow velocity waveforms in limb arteries, the most prominent consequences being the dicrotic notch and early diastolic flow reversal. Reflected waves have synchronous but opposite effects on blood pressure (augmentation) and flow (decrease) waveforms, which explain most of the differences in shape between arterial pressure and flow waveforms [5]. Although the constant and unambiguous downstream consequence of an arterial stenosis is a decreased early systolic acceleration, the concomitant changes in the shape and duration of the systolic peak are complex due to the number of potentially conflicting involved mechanisms [7]. For instance, the downstream effect of a stenosis is a reduction in the amplitude of the pulse wave, resulting in lower pulsatility, together with distal vasodilation, which also decreases flow pulsatility and attenuates wave reflection. However, arteriosclerosis, which concerns the whole arterial system, contributes, in combination with aging and hypertension, to an increase in pulse pressure and wave reflection since arterial wall stiffening hampers the buffering (“windkessel”) function of large arteries [8,9]. Therefore, only the first part of the systolic upslope (i.e., early systolic acceleration) is directly and proportionally related to the degree of upstream stenosis, whereas the amplitude and timing of reflected waves may result in a complex and ambiguous change in the shape and duration of the systolic peak.

However, although its direct measurement is possible, systolic acceleration is, in most cases, only indirectly estimated by measuring the duration of the rising part of the systolic peak, i.e., the time during which the blood flow propelled in the arteries by left ventricle contraction accelerates.

## 2. Measurement Methods

Current duplex and color Doppler ultrasonographic machines perform real-time Doppler frequency spectrum analysis and display waveforms that represent blood-flow velocity changes with time. From these waveforms, velocity (in m.s^−1^) and time (in ms) variables can be obtained, and indices can be calculated to characterize blood flow and detect upstream or downstream arterial obstruction.

Acceleration time (AT), measured in either the dorsalis pedis artery or the lateral plantar artery, has demonstrated a good to excellent correlation with both ankle-brachial pressure index in non-diabetic patients [10,11] and toe-brachial pressure index in patients with proximal, occlusive PAD [12]. With a cut-off value of 215 ms, AT allows for the detection of toe blood pressure ≤ 30 mmHg (one of the hemodynamic criteria of critical limb ischemia) with a 86% sensitivity and a 97% negative predictive value [12]. AT has also been reported as the most promising DW parameter for the prediction of femoropopliteal artery lesions: Yagyu et al. showed that a common femoral to popliteal artery AT ratio of 1.25 or more was highly indicative of stenosis greater than 50% (confirmed by angiography), with a 86% sensitivity, a 92% specificity, and a 0.93 c-index [13].

**Figure 1:**
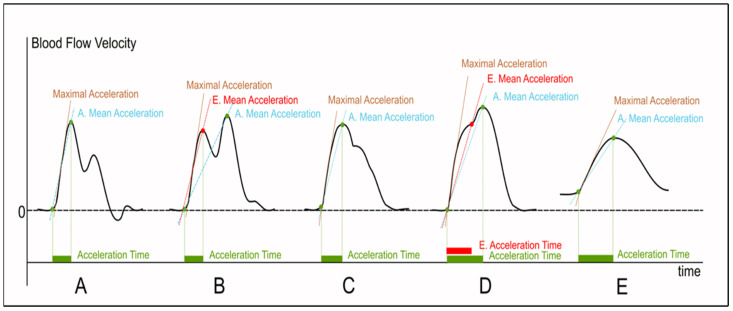
Methods of measurement of acceleration and acceleration time (also called systolic rise time) on Doppler flow velocity waveforms with normal and abnormal patterns. A. Mean Acceleration–Apex mean acceleration; E. Acceleration Time–Early peak acceleration time; E. Mean Acceleration–Early peak mean acceleration. A–Normal arterial waveform of triphasic waveform, B–increased reflected wave resulting in a bimodal pattern of a monophasic pattern, C–incident (early) and reflected (late) waves merging together on a monophasic pattern, D–inflection point on the upslope on a monophasic pattern, and E–abnormal monophasic flow pattern downstream from a stenosis, with dampened systolic peak.

AT was first proposed by Arima et al. in 1982 as a prognosis factor of renal allograft chronic rejection [3]. In 1988, Handa et al. proposed AT for the non-invasive diagnosis of renal artery stenosis, reporting that a cut-off value of 70 ms was highly predictive of a renal artery diameter reduction of 50% or more [14,15]. In these seminal studies, AT was described, with somewhat different wordings, as the time (in milliseconds) from the start of the systolic upslope to the apex of the systolic peak [10,11,12,13]. Unfortunately, referring to acceleration time or acceleration with vague definitions can be confusing [16]. Handa [15] defined AT as “the time period between the onset and peak frequency of the time-velocity spectrum”, referring to Arima et al. who provided only a graphical description [3]. In his PAD study in 1980, Humphrey et al. referred to the rise time (RT), defined as the “time period between the onset and peak forward frequency of the waveform”, and to the rise time ratio defined as the distal RT divided by the proximal RT ($\frac{distal\;RT}{\;proximal\;RT})$ [2]. Therefore, RT is basically identical to AT, although it does not explicitly refer to blood-flow acceleration. More recently, Yagyu et al. used the AT (defined as “the time from systolic acceleration to peak flow”) and AT ratio for the diagnosis of aorto-iliac and femoropopliteal stenosis [13]. The AT ratio was defined as the AT at the popliteal artery divided by the AT at the common femoral artery ($\frac{AT\;at\;popliteal\;artery}{AT\;at\;common\;femoral\;artery})$. To our knowledge, Strosberg et al. were the first to mention a more precise definition of the AT: “AT was manually measured by placing a caliper on the level at which the gradient begins to rise at the end of diastole to the first peak of systole (early systolic peak)” [17] (Figure 1A). The apex of the systolic peak is indeed quite clearly defined in normal time–velocity waveforms, e.g., in a triphasic or biphasic DW [8]. However, the apex becomes more and more blurred as the systolic peak becomes dampened and flattened, as in patients with PAD. Moreover, the apex corresponds to the first (or early) systolic peak in normal arterial waveforms. However, in patients with increased arterial wall stiffness due to aging, atherosclerosis, and/or arterial hypertension, the late systolic peak (related to pulse wave reflection) occurs earlier, thus heightening the apex (the so-called pulse “augmentation”) (Figure 1B,C) or even altering the systolic waveform pattern by introducing an inflection (or “shoulder”) on the uprising slope, as illustrated by Nishihira et al. and Iizuka et al. (Figure 1D) [18,19]. These authors explained that AT should be measured from the foot to the apex in the case of a monomodal systolic pattern (Figure 1A,E), measured to the first peak in case of bimodal pattern (Figure 1B), and to the inflection point if present on the upslope (Figure 1D) [18,19]. However, the early and late systolic peaks may merge together, and/or the inflection point may be poorly discernable. Moreover, due to the complex interplay between arterial diameter, blood viscosity, arterial wall stiffness, reflection sites, heart rate, left ventricular contractility, and downstream impedance [5,20], waveform changes can be equivocal. Most authors are probably measuring the AT from the foot to the apex whatever the pattern, but doubt remains when they fail to provide a detailed description of the method they use, since results can be quite different depending on the method and the flow pattern [18,21] (Figure 1).

**Figure 2:**
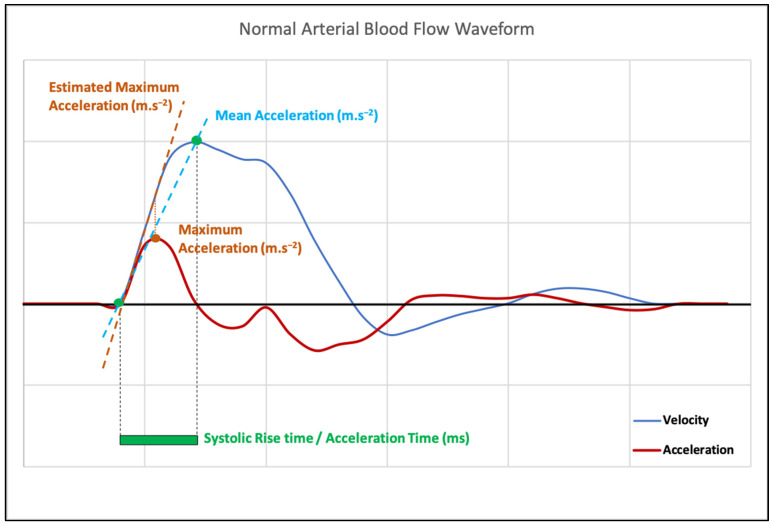

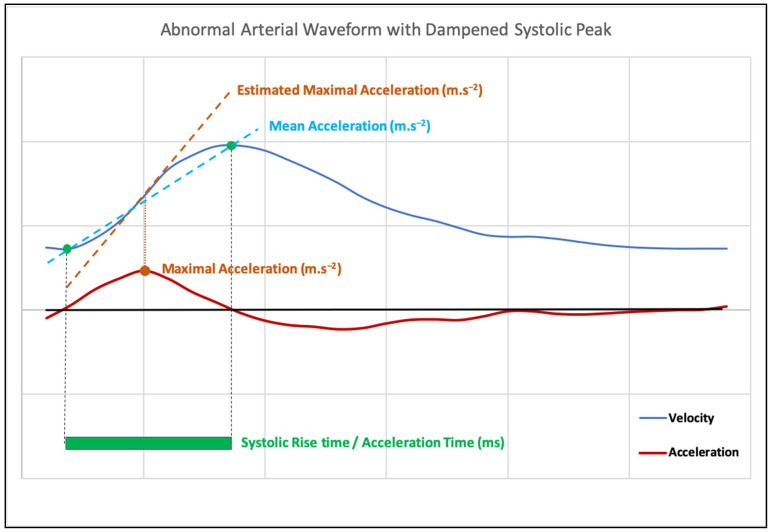
Time–velocity and time–acceleration curves in a normal, lower-limb artery and downstream from a significant stenosis with acceleration measurements.

Measuring AT (also called systolic rise time by some authors), i.e., from the foot to the apex, is straightforward, though sometimes imprecise. AT, in its simplest definition, is arguably the most convenient variable for routine assessment. However, measurements referring to the first part of the upslope, i.e., to the maximal acceleration, yielded better results for the detection of carotid and renal artery stenosis [18,21,22,23,24]. Maximal acceleration, at the very beginning of the systolic upslope, is not affected by reflected waves and other confounding factors. It is, therefore, more precise and reliable for the diagnosis of stenosis, but its measurement would require insonation angle correction and dedicated tools that are not currently available on every ultrasound machine. Accurate and reliable acceleration measurements, in m.s^−2^, are possible only if an <60° insonation angle can be obtained and precisely measured. Conversely, AT measurements are not dependent on the insonation angle. Maximal acceleration can be approximated as the slope of the hand-drawn straight line tangential to the steepest part of the systolic peak upslope on the time–velocity Doppler spectrum display (Figure 1). Nevertheless, this measurement is very operator-dependent, especially in pathological cases when the upslope is no longer virtually rectilinear (Figure 1E) [8]. Drawing a straight line between the foot and the apex of the systolic peak would estimate the mean acceleration, as described by Brouwers et al., but it would suffer from the same limitation as AT (Figure 2) [23,24]. On the other hand, maximal acceleration corresponds to the steepest part of the systolic upslope of the time–velocity curve or, as it was more precisely explained by Brouwers et al., [24],“the inflexion point at which upstroke changes from concave up to concave down”. It can be easily identified on the time–acceleration curve (the first derivate of the time–velocity curve) as the point at which acceleration changes from increasing to decreasing (Figure 2). Bardelli et al. suggested measuring the maximal acceleration as the slope of the early systolic phase, before the inflexion point if it is present [21].

Acceleration time (or systolic rise time), mean acceleration, and maximal acceleration yield similar results in normal arteries, but can be markedly different in abnormal arteries with a dampened systolic peak (Figure 2).

Moreover, inter-rater reproducibility seems weaker for the acceleration and acceleration time when compared to more standardized variables, such as the resistance index [16] or peak systolic velocity [19]. This may be partly explained by the lack of a measurement standard. Acceleration time and maximal acceleration measurement issues would be easily overcome by using the time–acceleration waveform, which is the first derivative of the time–velocity waveform, rather than the time–velocity waveform itself. Thus, AT would be the time between the two null values of the time–acceleration curve, before and after its maximum, respectively (Figure 2).

Whatever the variable (time or acceleration), absolute values (in ms or m.s^−2^) could be affected by cardiac disorders, e.g., heart failure or aortic valve stenosis [21,25,26]. Consequently, ratios (distal/proximal value, e.g., renal/aortic) should logically be preferred [2,18]. Additionally, the Ankle–Brachial Index (ABI) allows for the abstraction of heart pathology. We can imagine a pedal-radial index or pedal-aortic index, which could be the division of the AT in the pedal artery by the AT in the radial artery or aorta, respectively. However, increasing the number of variables, each of which has its own inherent variability, may impact the diagnosis reliability. Therefore, averaging a greater number of measurements would be mandatory when using ratios.

## 3. Proposal and Perspectives

**Figure 3:**
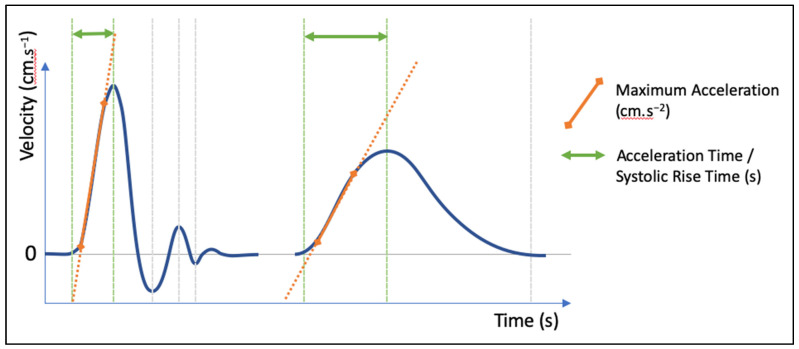
Proposal of standardized measurements for acceleration time (or systolic rise time) and maximal acceleration in normal (triphasic) et abnormal (monophasic) Doppler waveforms.

Considering the growing interest in systolic acceleration to assess lower-extremity PAD, we believe that some clarification would be useful [10,12,13]. Regarding the naming of measured variables, “acceleration” should be used only for variables directly and unequivocally related to flow acceleration and reported in m.s^−2^. Other variables related to the time duration of a part or totality of the systolic peak upslope should be labelled “time” and reported in ms. Maximal acceleration and acceleration time (or systolic rise time) are not equivalent. The time duration of the maximal acceleration phase of the systolic peak upslope should not be referred to as acceleration time or systolic rise time since these variables are quite distinct, and the more severe the PAD, the greater the difference between them (Figure 3). For the sake of clarity and reliability, only two variables should be measured in future research studies, namely AT (or systolic rise time, in ms) and maximal acceleration (in cm.s^−2^). Additionally, the scrolling speed (in mm/s) of the spectral Doppler waveform should be specified (Figure 3). In lower-limb arteries, AT should be measured from the point where the acceleration becomes positive at the end of the diastole (beginning of the velocity curve upslope) to the point where the acceleration becomes negative for the first time (top of the first systolic apex). Unless an automatic measurement function using the first derivative of the time–velocity curve is implemented in the US machine, we recommend that maximal acceleration should be measured as the slope of the hand-drawn straight line tangential to the steepest part of the systolic peak upslope. Considering that both the AT and acceleration measurements are thus, in most cases, performed manually, and as such are subject to intra-observer variability, we recommend that at least two distinct measurements should be performed on each DW and averaged.

## 4. Conclusions

When referring to the acceleration time or systolic rise time (in ms), authors should precisely state their measurement method. Taking into account the waveform pattern and measuring from the foot to the early systolic peak or to the inflection point rather than to the apex is preferable, but sometimes difficult. Theoretically, maximal acceleration (in cm.s^−2^) is a more accurate variable as it is not affected by wave reflection; however, its manual measurement on the time–velocity curve is very operator-dependent and poorly reliable when the systolic peak is dampened. Mean acceleration is easier to measure but suffers from the same pitfalls and limitations as the AT and provides no additional information. In most, if not all, duplex Doppler systems, real-time Doppler frequency spectrum analysis offers automatic calculations and a display of the mean and maximum flow velocity. From the maximum velocity waveform, sonographers should calculate the first derivative, thus providing actual and accurate measurements of the maximal acceleration. In the meantime, further research is needed to determine, in the clinical routine, which of these measurements (maximal acceleration or acceleration time) would provide the best accuracy and reproducibility in patients with PAD.

## Data Availability

Not applicable.
